# Predicting the transport of 2,4-dinitroanisole (DNAN) and 3-nitro-1,2,4-triazol-5-one (NTO) in sandy and sandy loam soils

**DOI:** 10.1016/j.heliyon.2022.e11758

**Published:** 2022-11-22

**Authors:** Encina Gutierrez-Carazo, James Dowle, Frederic Coulon, Tracey Temple, Melissa Ladyman

**Affiliations:** a Cranfield University, Centre for Defence Chemistry, Defence Academy of the United Kingdom, Shrivenham, SN6 8LA, UK; b Golder, Sirius Building, The Clocktower, Edinburgh, EH12 9LB, UK; c Cranfield University, School of Water, Energy and Environment, Cranfield, MK43 0AL, UK

**Keywords:** Environmental impact, Insensitive high explosives, Soil contamination, GoldSim, Monte Carlo method

## Abstract

There is a trend toward the use of Insensitive High Explosives (IHE) in both military and civil applications as they are intended to be less prone to accidental detonation compared to traditional explosive fills. This has driven the development of new explosive formulations containing different chemical compounds whose behaviour once they are released into the environment is not fully understood. To date, research into the toxicity and the persistence of IHE compounds in the environment is scarce and little has been described about how they interact with, or move through soil. In this work, the transport of two IHE constituents, 2,4-dinitroanisole (DNAN) and 3-nitro-1,2,4-triazol-5-one (NTO), through two soil types (sand and sandy loam) was simulated in GoldSim using a stochastic approach. The simulation outputs were validated by comparison to results from empirical soil column experiments.

Sorption of the IHE constituents to the soil was the most significant factor in predicting when the contaminants eluted from the soil column. Sensitivity analysis demonstrated that variation in the matrix water partition coefficient (K_d_) had the greatest influence when used to predict the IHE compounds transport. K_d_ was measured empirically and, as expected, it was low in sand for NTO (0.334 L kg^−1^) and DNAN (0.401 L kg^−1^), suggesting high mobility. While in sandy loam K_d_ for NTO (0.242 L kg^−1^) was similar to one obtained in sand, it was significantly higher for DNAN (9.128 L kg^−1^), explaining the high retention and adsorption in the sandy loam soil. The use of stochastic modelling to estimate IHE breakthrough concentrations could enable the uncertainty inherent in environmental systems to be embedded into simulations, thus increasing their representativeness. This study is the first step toward proactive management of IHE in the environment, and may support decision making for remediation and mitigation strategies in different environments.

## Graphical abstract

**Figure:**
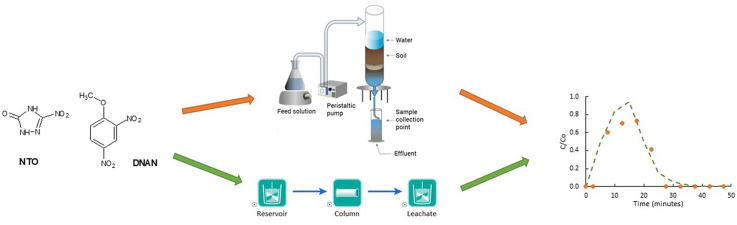

## 1 Introduction

For decades, explosives have been used for defence and industrial purposes (Mark et al., 2016; Walsh et al., 2018). Activities involving the manufacturing of these materials and their disposal by open-burning, as well as second order detonation and blow-in-place detonations have been demonstrated to deposit up to 50% of the explosive on the soil surface leading to potential contamination of nearby water and soil (Temple et al., 2018). Explosive compounds can be toxic to bacteria (Madeira et al., 2018), animals (Lotufo et al., 2015) and plants (Vila et al., 2008) and therefore, it is essential to manage their environmental impact to preserve ecosystems (Beck et al., 2018). In recent years, the drive for less sensitive explosives i.e. those less likely to be detonated by accidental insult, has seen the replacement of traditional explosive fills by Insensitive High Explosive (IHE) fills. This new generation of explosives uses mixtures of organic compounds such as 2,4-dinitroanisole (DNAN) and 3-nitro-1,2,4-triazol-5-one (NTO) in combination with traditional explosives to meet the insensitivity and efficiency requirements. However, these new chemical compounds have different physicochemical properties compared to traditional explosives. Specifically, NTO is significantly more soluble in water than legacy explosives (12.6 g/L, 25 °C), has three different tautomeric structures, is acidic (pKa = 3.76) and sensitive to biodegradation (Patent No. 9165, 1988; Madeira et al., 2018; Temple et al., 2018; Wei et al., 2021). Due to this, NTO is likely to rapidly transport through the environment, although it can degrade when soils are highly rich in organic matter (Mark et al., 2016). In contrast, DNAN exhibits low sensitivity and has been used in cast formulations to replace TNT (Deng et al., 2021). It is moderately soluble (276 mg/L, 25 °C) and is less toxic to mammals than traditional explosive fills, though may undergo biodegradation to more toxic species (Davies and Provatas, 2006; Dontsova et al., 2014; Kitcher et al., 2017). These physicochemical properties influence DNAN and NTO transport or movement through the soil matrix, increasing processes such as adsorption, interaction with the organic matter, degradation and plant uptake compared to legacy explosive compounds. Hence, the physicochemical properties of the chemicals and the soil matrix in combination with the environmental conditions determine the mobility of NTO and DNAN in soil. Nevertheless, the current knowledge of IHE toxicity and their persistence in the environment is not sufficient for risk assessment and remediation strategies for contaminated land. This raises questions about how these IHE can be studied in the environment, and what are the most effective methods for assessing their environmental impact.

Often, explosive transport in soil is investigated by performing controlled laboratory experiments attempting to replicate processes that occur in the real environment (Kalderis et al., 2011). These experiments can be classified into static, in which the outcome does not change over time, and dynamic, in which the outcome changes over time (UK Government Office for Science, 2018). Static experiments, such as bottle flask tests and microcosms have been performed to study processes affecting persistence such as the degradation rate of explosives and reversible and irreversible sorption onto soil (Halasz et al., 2018; Indest et al., 2017; Krzmarzick et al., 2015; Won and Borden, 2017). In this way, key parameters for defining transport processes such as partition coefficients (matrix-water, organic carbon-water, octanol-water etc…) and half-life times of IHE in different media have been quantified. However, some of these parameters are highly dependent on the soil type and the environmental conditions and therefore cannot be extrapolated to other environments.

Soil column and lysimeter indoor experiments can be used as dynamic systems to study rate of explosive transport in the environment (Arthur et al., 2018; Heerspink et al., 2017; Molina et al., 2006; Temple et al., 2018). In these studies, processes such as explosive breakthrough in different soils have been investigated with the aim to determine their persistence and potential degradation in the soil. However, experiments are often only conducted with the soil type, contaminant and environmental conditions of interest making it challenging to draw broad conclusions for specific contaminants. Therefore, full investigation of a single contaminant can be expensive and time consuming, which has motivated the scientific community to search for alternative, non-experimental methods for determining the key parameters for explosive fate and transport.

To overcome these limitations, the development and use of computational methods to study environmental impact by simulating fate and transport mechanisms has increased during the past two decades (Beck et al., 2018). The principal advantages of computational methods are shorter time period, lower cost and the ability to study environments without needing to use potentially harmful contaminants. Computational methods for studying contaminant transport can be divided into either deterministic or stochastic approaches. Deterministic models give the same single output once the inputted data is set, and, although they often have a higher uncertainty and the simulations obtained with this approach, they are usually not sufficiently representative of the real environment. Conversely, a stochastic approach has an inherent element of random or uncertain behaviour and the events are assigned probabilities i.e. the outcomes are expressed as a distribution of probabilities instead of a single value. This second approach is suitable for exploring patterns, assessing the probability of the outputs, and completing datasets or situations with high uncertainty (Kirchsteiger, 1999).

One such software is GoldSim, which is known for its incorporation of the Monte Carlo method (GoldSim Technology Group, 2018), enabling the representation of uncertainties by using a probabilistic approach and facilitating simulations in complex systems. GoldSim has been used to simulate transport of radionuclides (Panik and Necas, 2013), inorganic compounds (Lu et al., 2012), and water management systems (Bhuyian et al., 2015) in the environment. Regarding organic contaminants, Goldsim has successfully been used to determine the distribution and fate of aliphatic and polycyclic aromatic hydrocarbons in chalky soils (Cao et al., 2015), which suggest that this software could potentially be used to simulate alternative organic chemicals in the soil.

However, there have been no published studies about the use of GoldSim to simulate explosive transport. GoldSim incorporates a Contaminant Transport module, specific for the study of contaminants in different media (solid, water and air) and along with the Monte Carlo simulation, which makes it a suitable computational program for modelling explosive transport in soil.

Due to the complex interaction of soil characteristics and chemical properties that determine the persistence of contaminants in the environment, it is very challenging to experimentally determine the potential risk of explosive mixtures in the environment. Computational methods present an opportunity to overcome the challenges of laboratory experiment, however very few models have been developed to simulate the persistence of explosives in the environment. Explosive mixtures can result in complex environmental systems that contain a large number of variables such as climate, soil type and geomorphology, making it extremely challenging to predict their mobility in the soil system. Therefore, there is a need to develop effective methods for environmental practitioners and risk managers to predict the fate and transport of these chemicals to evaluate the risks posed by explosive residues and to mitigate their impact in the environment.

The aim of this work was to develop a representative model using the GoldSim simulation environment to study the mobility of NTO and DNAN in two soils with differences in organic matter content and particle size. To test the model, breakthrough times and maximum concentration in leachate simulated by GoldSim were compared to the same data collected from controlled laboratory soil columns replicating the simulation. This also enabled the identification of essential parameters required to produce a representative model that could be used to aid environmental risk assessment and remediation strategies for IHE. Ultimately, this work introduces a novel and user-friendly simulation framework to be used by practitioners rather than simply for academic purposes.

## 2 Materials and methods

### 2.1 Soil preparation and characterisation

Two soils were purchased from a building merchant in Oxfordshire (UK), classified as ‘sandy loamy’ and ‘sandy’ soils by their particle size distribution. Quartz sand was purchased from Fisher Scientific UK and used as received. Soils were air-dried for 2 weeks and passed through a 2 mm sieve to remove the largest fractions. The particle size distribution was determined according to ASTMD2487-11, which classified the two soils as sand and sandy loam. Soil moisture (BS 13772:1990), pH and total organic content (BS 13039:2001) were determined according to British Standards. Total Carbon Nitrogen (TCN) content was determined using Elementar Vario ELIII (Table 1) (Temple et al., 2018).

**Table 1:** Characterisation of quartz sand, sandy and sandy loam soil 1. Determined by incineration at 400 °C for three days; 2 Determined by griffin pH meter model 80 at room temperature; 3. Determined by Elementar Vario ELIII.

Soil	Description	Organic matter content^1^ (%)	pH^2^	Mode particle size (mm)	TCN (%)^3^		
					N	C	C/N
Quartz sand	100% quartz, inert material	-	7.1		-	-	-
Sand soil	UK sandy	4.9	7.9	0.2–0.6	0.012	7.22	601
Sandy loam soil	UK topsoil	8.0	8.0	0.6–2	0.30	5.11	17

### 2.2 Preparation of IHE compound stock solutions

DNAN was used as purchased from SIGMA (98%). NTO was synthesised in house (Lee and Coburn, 1988 and Sandham et al., 2013). For the column transport studies stock solution DNAN (∼40 mg) and NTO (∼40 mg) were solubilised in distilled water (1 L) at room temperature for 48 h.

100 mg L^−1^ stock solutions of DNAN and NTO were used to prepare lower concentration solutions (5, 10, 20, 30 and 40 mg L^−1^) for the batch soil/water partition coefficient studies. These solutions were also used as High Performance Liquid Chromatography (HPLC) standards.

### 2.3 Determination of soil water partition coefficient (Kd)

Samples of quartz sand, loamy or sandy soil (∼5 g) were submerged in an aqueous DNAN and NTO solutions at 5, 10, 20, 30 and 40 mg L^−1^ in 100 mL Erlenmeyer flasks. The flasks were wrapped in foil to avoid UV exposure and shaken for 18 h at 150 rpm, ambient temperature and pressure (Arthur et al., 2017). Supernatant was removed with a micropipette and stored at 4 °C until analysis by HPLC. A mixture of acetonitrile (ACN):water (1:1) (10 mL) was added to the soil and shaken for another 24 h at 150 rpm to extract NTO and DNAN retained in the soil (T. . Temple et al., 2019). The supernatant was then removed and stored at 4 °C until analysis by HPLC. The supernatant and extract concentrations were determined by HPLC. Experiments were performed in triplicate for DNAN and NTO in each soil.

Water soil partition coefficient was determined by Eq. (1):

(1) $$K_{d}={\left[A\right]}_{soil}\;/\;{\left[A\right]}_{water}$$

Where [A]_soil_ is the contaminant concentration the soil and [A]_water_ is the contaminant concentration in water, that is, the section of the soil that is covered by water (Borgesen et al., 2015).

Batch experiments were performed in triplicate to determine the NTO and DNAN K_d_ in sandy and loamy soils. Partition coefficient values for NTO and DNAN described in the literature varied greatly depending on the soil or matrix used supporting the conclusion that experimentally derived values for the specific soil-contaminant system of interest were essential (Arthur et al., 2018; Mark et al., 2017) (Table 2).

**Table 2:** Water-soil partition coefficient (K_d_) and standard deviation for DNAN and NTO in sandy and loamy soil in triplicate.

Compound	Soil	K_d_ (L kg^−1^)	Standard deviation (L kg^−1^)
NTO	Sand	0.334	0.025
	Loam	0.242	0.170
DNAN	Sand	0.401	0.082
	Loam	9.128	1.505

### 2.4 Soil columns

Three glass columns (2.5 cm × 10 cm) were filled with dried and sieved quartz sand, sandy or sandy loam soil in triplicate (270.7 ± 0.1 g, 275.6 ± 0.1 g and 195.9 ± 0.1 g respectively) and gently compacted with a flat ended tool until no visible gaps were observed (Dontosova et al., 2006; Temple et al., 2018). The columns were saturated with distilled water (up to 100 mL) and allowed to settle for one hour. DNAN or NTO stock solution (40 mg L^−1^) was added in 50 mL increments every 5 min for 15 min, followed by 40 min with distilled water influent at 10 ml min^−1^(Kramoer Dosing Pump, China). The total time was 55 min and leachate was collected every 5 min. Samples were filtered with 0.2 μm polyethylene terephthalate (PET) filters and stored at 4 °C, until analysis. Analysis was normally done within 2 days.

### 2.5 High performance liquid chromatography coupled with diode array detector

HPLC was performed using a Waters-Alliance 2696 equipped with a Waters 996 photodiode array detector (USA). The analytes were separated on a ZORBAX Eclipse Plus C18 column (4.6 × 150 mm, i.d. 3.5 μm) from Agilent Technologies (Wilmington, DE, USA) maintained at 30 °C. Samples were injected with a syringe loading injector fitted with 10 μL loop. Optimum chromatographic conditions were obtained with a linear gradient of 40% ACN (solvent A) and 60% water acidified 0.1% with formic acid (solvent B) with a flow rate of 1.5 mL min^−1^. The analytes were quantified via UV absorbance with optimum sensitivity detected at 296 nm for DNAN and 315 nm for NTO. Retention times were 1.01 min for NTO and 4.96 min for DNAN. The calibration curve was obtained by plotting the concentration against corresponding peak area for each analyte and both peak areas of samples and standards were determined by Empower 2 software (Waters). For this method, limit of detection was 0.5 mg L^−1^ and limit of quantification was 1.4 mg L^−1^ for both DNAN and NTO (Fawcett-Hirst et al., 2020; Temple et al., 2018).

### 2.6 GoldSim simulations

GoldSim version 12.1 and the Contaminant Transport Module were used, with the modelling primarily using the Material, Pathway, Selector as well as Input and Result elements (GoldSim Technology Group, 2018). The properties of the different soils, water and contaminants were defined in the element of Material which includes the species, water and the solids used for modelling. In the Species element the molecular weight of DNAN and NTO (198 and 130 g mol^−1^ respectively) was specified. Quartz sand, sandy soil and sandy loam soil were considered the ‘Solid’ and therefore actual bulk density and porosity were inputted from experimental measurements in triplicate (Table 3). Tortuosity was estimated (Alaoui et al., 2003).

**Table 3:** Table with inputs for GoldSim. All of them were inputted as single (not distributed) and simulations have been done once.

Soil Type	Parameter	Value	Justification
Quartz sand	Bulk Density	1.38 g cm^−3^	Experimentally measured
	Porosity	0.36	Experimentally measured
	Tortuosity	0.6	Estimated
Sandy soil	Bulk Density	1.40 g cm^−3^	Experimentally measured
	Porosity	0.29	Experimentally measured
	Tortuosity	0.5	Estimated
Sandy loam soil	Bulk Density	1.09 g cm^−3^	Experimentally measured
	Porosity	0.44	Experimentally measured
	Tortuosity	0.5	Estimated

The model was constructed using basic initial inputs displayed as Data elements and Functions (Suppl. 1). The scenario started with an initial contaminant concentration that was spilled from a tank into the soil column, which is represented by a specific type of Pathway element called a Pipe. Influent (40 mg L^−1^ of NTO and 40 mg of DNAN) was simulated entering the Pipe to match soil column experiment influent rate. Each soil was defined as the Solid inside the Pipe and its main dimensions and properties of the Pathway were defined according to the parameters used in the laboratory soil column experiments (Table 4). The contaminant was added to the column as a continuous concentration of 40 mg L^−1^ for both DNAN and NTO using the option Input Rate. The continuous inflow and outflow passing through the soil column was 10 ml min^−1^ for both containers with contaminated and non-contaminated water.

**Table 4:** Pipe pathway properties used in GoldSim to model the dimensions of the soil column.

Parameter	Value	Justification
Length (cm)	10	Measured
Radius (cm)	2.5	Measured
Area (cm^2^)	12.57	Calculated
Volume (cm^3^)	196	Calculated
Perimeter (cm)	16	Calculated
Dispersivity (cm)	1	GoldSim help handbook

GoldSim simulated contaminant transport by using advection equation (Eq. (2))

(2) $$\frac{dC}{dt}=-v*\frac{dC}{dx}$$

Where t is time (min) v is the speed of the flowing water (mL min^−1^), A is the transversal area of the column (cm^2^), C is the contaminant concentration (mg L^−1^) and x is the distance along the column (cm).

It also used the partitioning governing equation (Eq. (3))

(3) $$\frac{dM}{dt}=Q*\frac{M}{V+K_{d}G}$$

Where M is the mass of contaminant (mg), V is the volume of water in the column (mL), K is the contaminant soil-water partition coefficient (L kg^−1^) and G is the soil mass in the column (kg), t is time and Q is the water floerate (mL min^−1^).

Last, to estimate contaminant travelling time, it included the retardation factor (R) of each contaminant in the column (Eq. (4))

(4) $$R=1+\frac{d*K_{d}}{n}$$

Where d is the soil density (kg L^−1^), Kd is the contaminant soil-water partition coefficient (L kg^−1^) and n is the soil porosity.

In addition, the Monte Carlo method was used to assess and address uncertainties and to understand the impact of the inputted parameters on the breakthrough curves. In this model, the matrix-water partition coefficient of contaminants in each soil was studied and represented by a stochastic input of uniform distribution indicating the maximum and minimum values. The rest of inputs used in the model remained invariable, so the variability in outcome was easier to visualize and identify. The simulation settings were modified, to allow for 101 realizations, as it was considered representative enough for assessing this model developed in GoldSim.

### 2.7 Sensitivity analysis

Sensitivity analysis was performed by making minor adjustments (slight variations) to the input values for bulk density, porosity, tortuosity and K_d_ and observing how NTO breakthrough varied. DNAN results for this sensitivity analysis were expected to be the same, as the model works with the same parameters for both chemical compounds. Sensitivity analysis was primarily conducted on sandy soil, as quartz sand is inert, and it is not representative of a real matrix. The input values varied in the analysis were chosen randomly, but realistically (i.e. a soil can never have a porosity of 100%) (Table 5). Small independent variations that made significant changes to the output of the simulation were consequently considered key parameters and were therefore determined experimentally for the specific soil and contaminant mixtures.

**Table 5:** Parameters used for the sensitivity analysis and their values.

Parameter	Values	Percentage Variation (%)
Bulk density (g/L)	1.20, 1.40, 1.60 1.80	60
Porosity	0.20, 0.25 0.30 0.35	75
Tortuosity	0.5, 0.6, 0.7, 0.8	40
Partition coefficient (l/kg)	0.0, 0.1, 0.2, 0.3, 0.4,	40

## 3 Results and discussion

### 3.1 Simulating NTO and DNAN transport in quartz sand

For first iteration of the DNAN and NTO GoldSim transport model only bulk density (1.38 g L^−1^), tortuosity (0.6) and porosity (40%) were inputted to GoldSim as calculated based on the equivalent laboratory quartz sand column. The only chemical property included in this version of model was the molecular mass of DNAN or NTO. The flow rate parameter for ‘Pipe’ element was 10 mL min^−1^ which was replicated in laboratory experiments by dosing the columns with 50 mL DNAN or NTO stock solution (∼40 mg L^−1^) every 5 min for 15 min, followed by pure water for 40 min to mimic a limited release of contaminant. The volume of influent was determined by the rate of transport of the waterfront in the slowest (sandy loam) soil column. To enable direct comparison between simulated and experimental results leachate concentration was normalised over the initial concentration (C/C_O_) and plotted against time.

**Figure 1:**
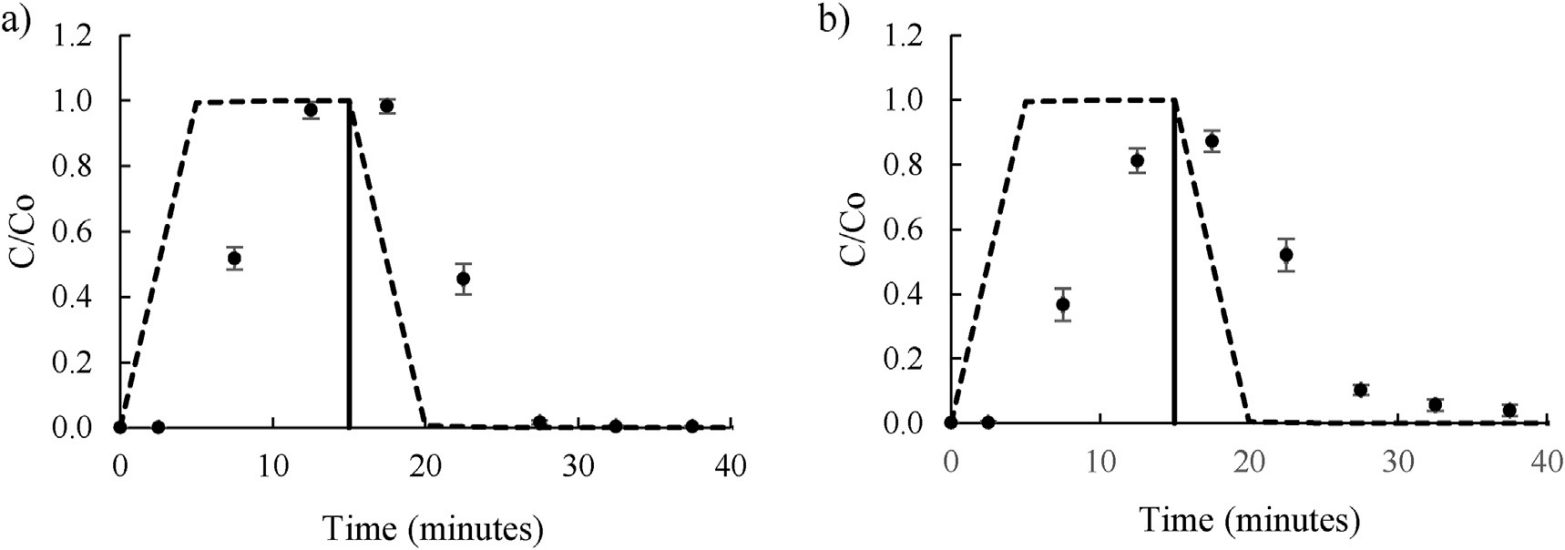
Comparison between the GoldSim simulation and laboratory experiment breakthrough curves for NTO (a) and DNAN (b) in quartz sand. The dots indicate experimental results and the discontinuous line is the simulated results from GoldSim. The solid line indicates time at which contaminated influent was exchanged for distilled water influent.

The initial GoldSim simulation compared well to laboratory column experiments with NTO (Figure 1a) and DNAN (Figure 1b) in quartz sand, an inert material used to determine the initial contaminant breakthrough with a non-interactive matrix. Experimentally, NTO concentration peaked between 15 and 20 min with the outflow concentration matching the inflow concentration and reaching 100%. DNAN concentration peaked between 15 and 20 min, although the highest recorded concentration was only 87% of the initial concentration. The total recovery of DNAN and NTO after passing through the quartz sand column were 105 and 104%, respectively, with the difference to GoldSim simulation (100%) account for by standard errors in the experimental procedure (e.g. peristaltic pump flow volume). Over the duration of the experiment, NTO and DNAN did not undergo physical nor chemical degradation nor irreversible immobilization to the matrix, which was expected for the quartz sand control.

The GoldSim simulation modelled similar peak breakthrough concentrations, estimating 100% for both DNAN and NTO. However, simulated contaminant breakthrough curves, particularly the gradients of increasing and decreasing DNAN and NTO concentration were not representative of experimental ones. The experimental breakthrough concentrations increased gradually to a peak, whereas breakthrough concentrations in GoldSim simulations plateaued at the influent concentration for ten minutes. Experimentally, increasing gradients were not as steep as those simulated by GoldSim (0.81 and 0.60 for NTO and DNAN, respectively compared to 1.37 for both simulated by the programme). Experimental decreasing gradients differed to those simulated by GoldSim more significantly (−0.67 and -0.46 respectively, compared to -1.37 for both simulated by GoldSim). The differences between the gradients may be due to the experimental set up, as the flow rate was slighlty variable when adding the contaminant solution to the column. In addition, due to the heterogeneity of the matrix and large particle size, DNAN and NTO infiltration may have followed preferential pathways in the matrix that could not be predicted by GoldSim. Therefore, GoldSim was not able to simulate the slight retardation of DNAN and NTO in quartz sand, simulating maximum concentration at only 5 min after the initial influent entered the column for both DNAN and NTO compared to 20 min experimentally. This implies that GoldSim assumes immediate contaminant transport to the bottom of the ‘Pipe’ and thus instantaneous appearance in the leachate.

It was possible to correct the retardation in breakthrough manually by experimentally measuring the time taken for influent to travel the length of the column during saturation and including this in the GoldSim model using the function element ‘Selector.’ This gave excellent comparability between simulated and experimental results for both DNAN and NTO in quartz sand. However, it was preferable to improve the model by including additional physico-chemical parameters of the soil and contaminants that fewer manual corrections would have to be made. Given the limited number of variables used to simulate the DNAN and NTO transport, the GoldSim transport simulation showed promising similarity to experimental results. However, it was preferable to improve the model by including additional physico-chemical parameters of the soil and contaminants that fewer manual corrections would have to be made. Therefore, to further develop the GoldSim DNAN and NTO transport model the inclusion of additional parameters for more complex soils was investigated.

### 3.2 Simulating NTO and DNAN transport in soil (sand and sandy loam)

**Figure 2:**
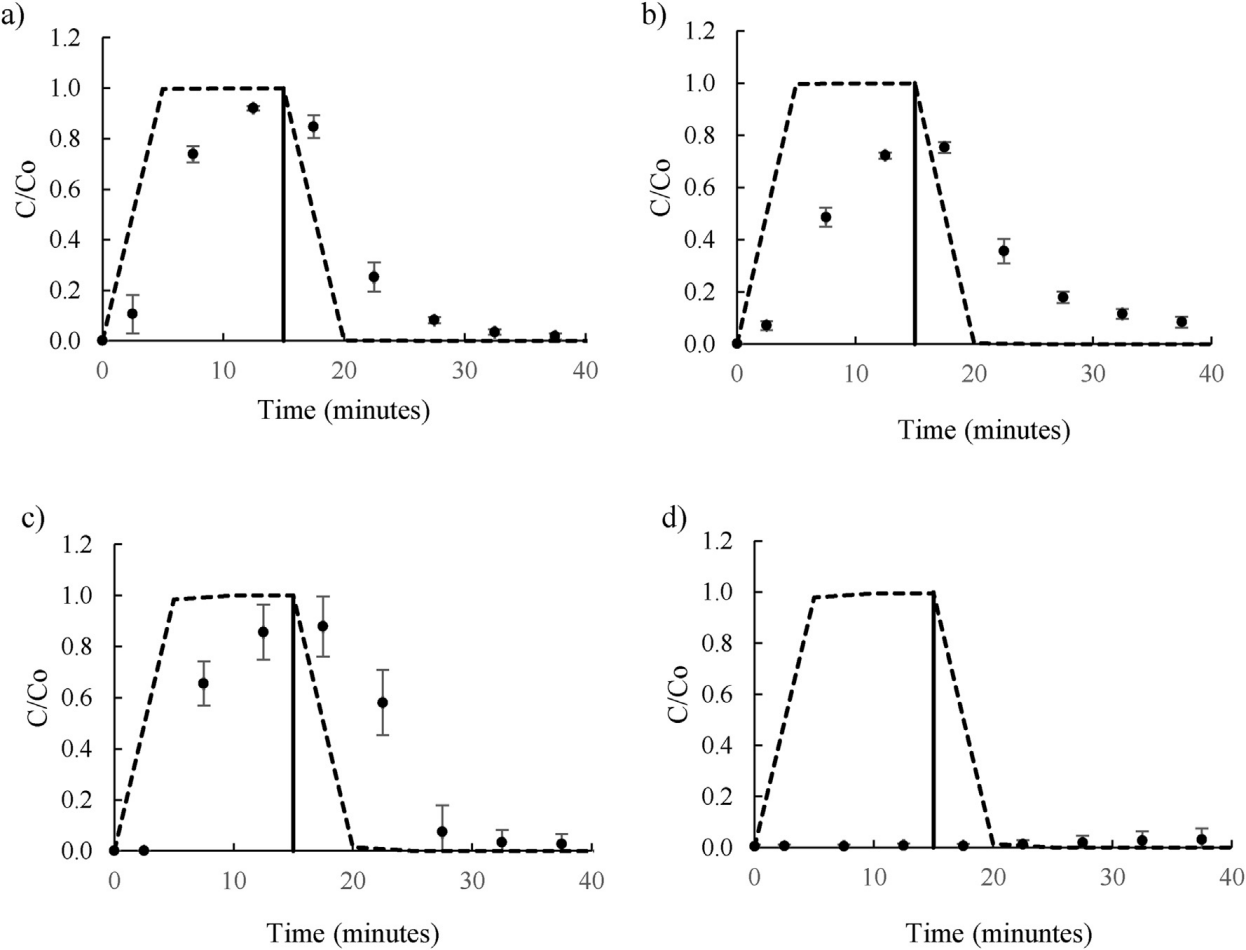
Comparison between the GoldSim simulation and laboratory experiment breakthrough curves for NTO (a) and DNAN (b) in sand, and (c) NTO and (d) DNAN) in sandy loam. The dots indicate experimental results and the discontinuous line is the simulated results from GoldSim. The solid line indicates time at which contaminated influent was exchanged for distilled water influent.

Two UK soils classified as sandy soil and sandy loam soil, were used to investigate the most influential parameters required to simulate the transport of DNAN and NTO. Both soils had similar pH, although they showed differences in their particle size and composition, with sandy loam soil containing a higher percentage of organic carbon (8.8%) compared to 4.9% for sandy soil, These two soils were chosen to increase the complexity of the simulation as DNAN adsorbs significantly to soils with high organic content, and NTO rapidly degrades (Arthur et al., 2018; Terracciano et al., 2018). The percentage of organic matter content is key for sorption processes, as organic particles in the soil can interact with DNAN and NTO by hydrogen bonding, covalent and donor/acceptor binding, polarity, and van der Waals forces (Kadoya et al., 2021; Mark et al., 2016). The nitro groups of the explosive compounds may promote polar interactions, as well as van der Waals forces between polar groups (such as –COOH, –OH and –NH) and aromatic groups found in organic matter particles (Vitale and Di Guardo, 2019). This promotes chemical sorption to the soil and retards its transport through soil matrices. The presence of organic matter and inorganic matter content, such as iron complexes in the soils, created more complex interaction between DNAN and NTO and the soil, difficult to simulate due to the simplicity of the model. To identify the key variables required for modelling these more complex scenarios, the simulation was initially run with the same GoldSim inputs as described in section 3.1, although soil inputted data elements were substituted by those obtained experimentally for the sandy and sandy loam soil: bulk density (1.40 g L^−1^ and 1.09 g L^−1^ respectively) and porosity (29% and 44% respectively). The other GoldSim data elements (such as molecular weight or explosive initial concentration) were kept the same. As before, both simulated and laboratory results were plotted as DNAN or NTO concentration in leachate divided by initial contaminant concentration over time (Figure 2a-d).

For the sandy soil, the NTO experimental concentration peaked between 10 and 15 min at 93% of the influent concentration (Figure 2a). DNAN concentration peaked between 15 and 20 min with a maximum concentration of 87% of the influent. However, the GoldSim model simulated the peak concentration for both DNAN and NTO to be after 5 min, the same as for quartz sand, suggesting that inputted parameters had the same drawback as in quartz sand simulations (Figure 2b). For comparison, sandy density and porosity were similar to quartz (varying only 8% and 2% respectively), thus, the simulations were expected to be similar disregarding all other interactions. Experimentally, total recovery of NTO and DNAN was 100 and 99% respectively, which matched GoldSim simulations, (100% after 55 min). Therefore, it is unlikely that degradation or irreversible immobilization of DNAN and NTO to the soil influenced the transport of these compounds in sandy soil.

As expected in sandy loam soil with higher organic content, the retention of DNAN was more significant than NTO. NTO peak concentration was detected between 15 and 20 min, five minutes later than in both quartz and sandy soil (Figure 2c). In addition, the peak concentration was only 72% of the influent concentration, compared to 100% as modelled by GoldSim. Experimentally, total NTO mass recovered from the soil was only 76% of the initial, indicating possible irreversible adsorption due to the interactions between the contaminant and organic matter. The DNAN simulation was even less representative for sandy loam soil as DNAN did not breakthrough the column at all, even though the GoldSim simulation estimated the first DNAN to be detected in the leachate after only 5 min suggesting that DNAN sorbed strongly to the soil. Although DNAN degrades rapidly in sandy loam soil (Temple et al., 2018), it is unlikely that significant degradation would have taken place within the timescale of the soil column (55 min) therefore the retardation of DNAN transport and low recovery in effluent is most likely due to adsorption (Figure 2d).

Simulations performed by GoldSim showed rapid DNAN and NTO elution time, while in reality it may take significantly longer for DNAN and NTO to elute from contaminated soil. Environmentally, this could mean the difference between a rapid influx of high concentration of contaminant that may cause acute toxic effects, or a lower, longer dose leading to more chronic effects (O’Doherty et al., 2013; Vila et al., 2008). Previous work has shown that adsorption of DNAN is much more rapid than adsorption of NTO, which is supported by the more significant retention of DNAN on sandy loam soil even within the short timeframe of the experiment (Arthur et al., 2018; Mark et al., 2016). It was evident that the initially developed GoldSim model was unable to simulate a representative breakthrough of NTO and DNAN in real soils with high organic content. Therefore, a sensitivity analysis was carried out to determine which key parameters had most influence on retention time and thus which parameters were essential to determine experimentally for the specific soil-contaminant combination.

### 3.3 Sensitivity analysis

**Figure 3:**
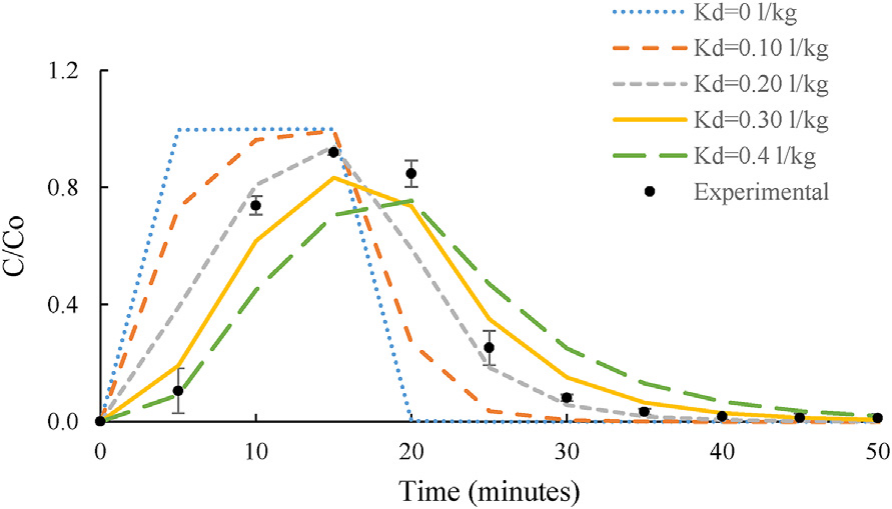
Sensitivity analysis. Variation of NTO breakthrough in sandy soil when modifying the value of NTO K_d_ in sand.

Sensitivity analysis was performed for the main three physicochemical parameters related to the soil already included in the model: density, porosity and tortuosity. These properties were chosen as they did not depend on the environmental conditions unlike flow rate or column dimensions but were integral to the system and vary depending on the matrix used. However, given the significant retention of DNAN and NTO observed in sandy loam soil it was evident that a parameter quantifying the retention was also required. Therefore, experimentally derived K_d_, was also included in the sensitivity analysis (Brannon et al., 2002; Yamamoto et al., 2004). Sensitivity analysis was undertaken for sandy soil and NTO among all the studied chemical-matrix combinations, as a representative system of how an explosive chemical would behave in the soil. Soil bulk density, porosity and tortuosity did not influence NTO breakthrough as changes to the inputted value of these parameters showed overlapping breakthrough curves (Suppl. 2). However, sensitivity analysis for the partition coefficient showed significant differences in peak breakthrough concentration time, with increasing K_d_ improving correlation to the experimental data (Figure 3). This was as expected as K_d_ tends to increase with increasing organic content, and the organic content of the sandy soil (4.9 %) was equivalent to literature values for higher partition coefficients. Therefore, it was concluded that an experimentally derived K_d_ for the specific soil-contaminant combination was essential for achieving more representative simulations.

Further simulations showed that when including K_d_ in the model, parameters such as density became more sensitive to the analysis i.e. altering the breakthrough curves. Density plays an important role in retardation, as packed soils usually retain a higher percentage of the contaminant and reduces the rate of contaminant transport (Dortch, 2018; Yamamoto et al., 2004). This reinforces the need to include experimentally derived K_d_s to the models created in GoldSim to achieve simulations as close to reality as possible. Therefore, K_d_ coefficients were obtained for DNAN and NTO in the two sandy and sandy loam soils for inclusion in the GoldSim model.

### 3.4 Simulating NTO and DNAN transport in soil using partition coefficient and Monte Carlo method

Experimental K_d_ values were determined by undertaking batch experiments (Mark et al., 2016) and were added to the ‘Solid’ element as a scalar vector of the element ‘Species’ (defined as DNAN and NTO). This input was added for each scenario as a ‘Stochastic element’ instead of a ‘Data element’ method, the input element used to add parameters in previous simulations. The stochastic element allows the definition of a maximum and minimum partition coefficient value instead of a unique value, allowing the user to assess the uncertainty of the breakthrough curve outcomes. The other inputs were the same as described in section 3.2 and were added as ‘data elements’ (single values). Both simulated and laboratory results were graphed with concentration of DNAN or NTO in leachate divided by contaminant initial concentration (C/Co) against time, including GoldSim breakthrough using maximum K_d_ and minimum K_d_. Minimum and maximum K_d_ were derived from the experimental average and its standard deviation.

**Figure 4:**
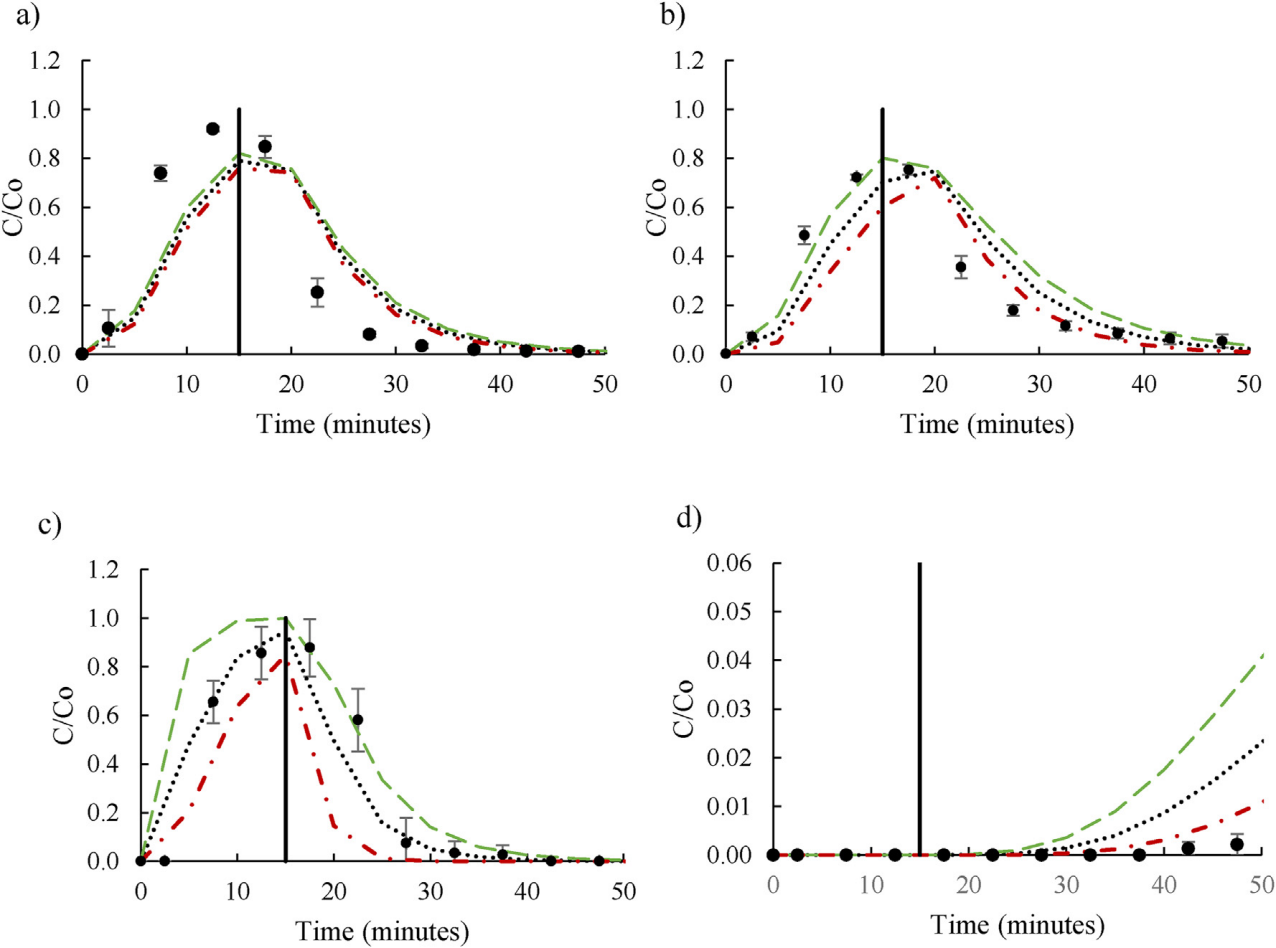
Comparison between the GoldSim simulation, including _Kd_ as a distribution element and 101 realizations using Monte Carlo, and laboratory experiment breakthrough curves for NTO (a) and DNAN (b) in sandy soil and NTO (c) and DNAN (d) in sandy loam soil. The dots indicate experimental results and the dashed lines indicate the simulated average (black), upper (green) and lower (red) limits. The solid line indicates time at which contaminated influent was exchanged for distilled water influent.

All previous simulations used deterministic methods, and produced one single output, which is unlikely to be a representative of the real environment. However, when including experimentally derived inputs with high standard deviations from experimental replicates, it is useful to demonstrate the variated scenarios. Using this probabilistic approach, the GoldSim simulation of breakthrough and peak concentration for NTO and DNAN transport was significantly more comparable to the experimental values in sandy loam and sandy soil when experimental K_d_ was included in the model (Figure 4a-d). The inclusion of K_d_ reduced the simulated length of time of peak concentration outflow from a plateau equal in time to the contaminant influent, to a peak that much more closely resembled the experimental data. In particular, the inclusion of K_d_ for DNAN persistence in sandy loam soil was able to simulate the complete retention of DNAN within the experimental timeframe.

Though similar, the simulation of NTO in sandy soil (Figure 4a) did not compare as well to the experimental values as the other simulations. Experimentally, peak breakthrough concentration was between 10 and 15 min, similar to GoldSim, which predicted a peak at 15 min. The maximum concentration was 90% of the initial experimentally compared to 87% in the model, which was within the margins of the experimental error. For DNAN, breakthrough peaked at the same time as simulation (between 15 and 20 min and 15 min respectively) at 78% and 80% of the influent concentration (Figure 4b). The addition of a variable K_d_ was clearly an improvement on the model as its simulations were more representative than those in section 3.2.

The standard deviations of experimentally derived K_d_ were higher in sandy loam soil than in sand, perhaps due to the higher organic content and therefore greater adsorption. In sandy loam soil, NTO experimental breakthrough concentration and time was within the simulated maximum and minimum breakthrough (Figure 4c). In the sandy loam soil, the DNAN K_d_ was very high indicating strong adsorption to the soil, explaining why inclusion of experimentally derived K_d_, increased the representativeness of the simulation. The simulation and experimental data matched well, with continuous retention of DNAN over time and no breakthrough of the contaminant (Figure 4d). Although the experimental breakthrough concentration was lower than the lower limit of the GoldSim estimated minimum breakthrough, the DNAN concentrations reported were so low that the difference is marginal. These results also agree with other studies in which DNAN was strongly adsorbed to soils rich in organic matter (Linker et al., 2015; Olivares et al., 2016; Todde et al., 2018). A possible explanation of the DNAN immobilisation to soil is due to the quinones contained in the humic acids which promote irreversible binding in aerobic conditions by strong covalent bonds (Beck et al., 2018; Deng et al., 2021). In addition, DNAN has been reported to undergo reductive transformation to aromatic amines inthe soil matrix (Kadoya et al., 2021). Other study reported that aromatic amines tend to covalently bind to carbonyl groups in natural organic matter by nucleophilic and possibly radical coupling reactions (Menezes et al., 2021). Although degradation processes have not yet been included in the model, it could explain the lack of experimental detection.

Overall, the simulations were significantly more representative of empirical results when K_d_ was included and the Monte Carlo method was used. Despite the short experimental timeframe (only 55 min in total), K_d_ is likely to be an essential parameter for simulating longer transport times as soil columns are dynamic systems in which the contaminant is in continuous movement, avoiding reaching equilibrium. However, the model poses some limitations for large-scale experiments, as parameters might vary and its simulations could not be validated by using laboratory facilities. For longer exposure experiments, only environmental inputs such as exposure time change, and K_d_ should be similar unless there is a drastic variation in environmental conditions such as temperature. The same would happen with larger soil columns, although here processes such as horizontal transport or matrix diffusion zones may participate more in the GoldSim model. K_d_ is, therefore, a key parameter for modelling DNAN and NTO transport in soils and it must be experimentally derived for the specific soil-contaminant matrix rather than estimated from literature to achieve representative results.

## 4 Conclusion

With the increasing drive for replacement of legacy explosive fills with IHE, it is likely that DNAN and NTO filled munitions will increasingly be used for training and defence purposes. Therefore, understanding the behaviour of these IHE in various environments prior to their extensive use is essential. The developed GoldSim transport model representatively simulated the transport of two key IHE components DNAN and NTO in two soil types using relatively few parameters. It was found through sensitivity analysis and comparison to laboratory experiments that the K_d_ was particularly important for representative simulation and must be experimentally determined for the soil-contaminant system of interest. Although it is necessary to experimentally determine key parameters for the system of interest for the model to be representative, these values are far quicker to obtain than running long environmentally representative laboratory experiments. In addition, the use of stochastic modelling enabled the uncertainty inherent in environmental systems to be embedded in the simulation further increasing its representativeness. The next stage will be to continue increasing the complexity of the system by testing larger systems, mixtures of soil types, mixtures of contaminants and different exposure scenarios e.g. IHE leaching from a solid mixture. However, the GoldSim model developed is the first of its kind to representatively simulate the transport of DNAN and NTO in real soils.

This study has meaningful applicability in the environmental management of IHE. First, the empirically determined soil and explosive physicochemical data can be used as inputs for models developed under similar conditions. For instance, K_d_ can be extrapolated to other studies with similar soil properties, avoiding the performance of tedious and time-consuming experiments. In addition, the outcome of the simulation can serve as a basis for other modellers to compare their simulations and make more representative predictions. Further to this, explosive breakthrough in the sand and sandy loam columns can be used to validate the simulations and performed by other modellers, which will be less time consuming and more economic in future research. Nonetheless, the most important applicability of the study relies on the probabilistic simulations, which can assist decision makers to evaluate the risks posed by IHE in the soil and to develop efficient remediation strategies in contaminated areas by IHE.

## Declaration

### Author contribution statement

Encina Gutierrez-Carazo: Conceived and designed the experiments; Performed the experiments; Analysed and interpreted data; Wrote the paper.

James Dowle: Contributed reagents, materials, analysis, tools or data.

Frederic Coulon: Conceived and designed the experiments; Analysed and interpreted data; Wrote the paper.

Tracey Temple: Conceived and designed the experiments; Contributed reagents, materials, analysis, tools or data; Wrote the paper.

Melissa Ladyman: Conceived and designed the experiments; Analysed and interpreted data; Contributed reagents, materials, analysis, tools or data; Wrote the paper.

### Funding statement

This work was supported by Engineering and Physical Sciences Research Council (18000030).

### Data availability statement

Data associated with this study has been deposited at

Sensitivity analysis.xlsx-https://doi.org/10.17862/cranfield.rd.20337168.

Experimental soil columns - https://doi.org/10.17862/cranfield.rd.20337165.

Simulations GS vs soil columns - https://doi.org/10.17862/cranfield.rd.20334591.

Soil-water partition coefficient - https://doi.org/10.17862/cranfield.rd.20310087.

Soil properties (Sept 2020) - https://doi.org/10.17862/cranfield.rd.20308932.

### Declaration of interest's statement

The authors declare the following conflict of interests: Tracey Temple and Melissa Ladyman are associate editors for Heliyon and Frederic Coulon is a section editor.

### Additional information

No additional information is available for this paper.

## Appendix A Supplementary data

Supplementary material- edited - clean version Supplementary material- edited - clean version

## References

[bib1] Alaoui A., Germann P., Jarvis N., Acutis M. (2003). Dual-porosity and kinematic wave approaches to assess the degree of preferential flow in an unsaturated soil. Hydrol. Sci. J..

[bib2] Arthur J.D., Mark N.W., Taylor S., Šimůnek J., Brusseau M.L., Dontsova K.M. (2018). Dissolution and transport of insensitive munitions formulations IMX-101 and IMX-104 in saturated soil columns. Sci. Total Environ..

[bib3] Beck A.J., Gledhill M., Schlosser C., Stamer B., Böttcher C., Sternheim J., Achterberg E.P. (2018). Spread, behavior, and ecosystem consequences of conventional munitions compounds in coastal marine waters. Front. Mar. Sci..

[bib4] Bhuyian, N. M., Thornton, J., & Kalyanapu, A. (2015). Developing “flood loss curve” for city of sacramento. 2015 ASFPM conference, 1–8. Retrieved from https://www.asfpmfoundation.org/ace-images/Developing_Flood_Loss_Curve_for_City_of_Sacramento.pdf

[bib5] Borgesen, C. D., Fomsgaard, I. S., Plauborg, F., Schelde, K., & Spliid, N. H. (2015). Fate of Pesticides in Agricultural Soils.

[bib6] Brannon J.M., Price C.B., Hayes C., Yost S.L. (2002). Aquifer soil cation substitution and adsorption of TNT, RDX, and HMX. Soil Sediment Contam.: Int. J..

[bib7] Cao X., Temple T., Li X., Coulon F., Sui H. (2015). Influence of particle size and organic carbon content on distribution and fate of aliphatic and aromatic hydrocarbon fractions in chalks. Environ. Technol. Innovat..

[bib8] Davies, P. J., & Provatas, A. (2006). Characterisation of 2 ,4-Dinitroanisole : an Ingredient for Use in Low Sensitivity Melt Cast Formulations (pp. 1–22). pp. 1–22.

[bib9] Deng Z. ying, Wang Y., Qi G. yu, Zhang Q. hua. (2021). High-pressure structural stability and melting performance of α-2,4-dinitroanisole. Ener. Mater. Front..

[bib10] Dontsova, K., Taylor, S., Pesce-Rodriguez, R., Brusseau, M., Arthur, J., Mark, N., … Šimůnek, J. (2014). Dissolution of NTO, DNAN and Insensitive Munitions Formulations and Their Fates in Soils. Retrieved from www.erdc.usace.army.mil.

[bib11] Dortch M.S. (2018). Modeling dissolution of high explosive formulations. Soil Sediment Contam..

[bib12] Fawcett-Hirst W., Temple T.J., Ladyman M.K., Coulon F. (2020). Adsorption behaviour of 1,3,5-trinitroperhydro-1,3,5-triazine, 2,4-dinitroanisole and 3-nitro-1,2,4-triazol-5-one on commercial activated carbons. Chemosphere.

[bib13] GoldSim Technology Group. (2018). GoldSim User Guide v12.1.

[bib14] Halasz A., Hawari J., Perreault N.N. (2018). New insights into the photochemical degradation of the insensitive munition formulation IMX-101 in water. Environ. Sci. Technol..

[bib15] Heerspink B.P., Pandey S., Boukhalfa H., Ware D.S., Marina O., Perkins G., WoldeGabriel G. (2017). Fate and transport of hexahydro-1,3,5-trinitro-1,3,5-triazine (RDX) and its degradation products in sedimentary and volcanic rocks, Los Alamos, New Mexico. Chemosphere.

[bib16] Indest K.J., Hancock D.E., Crocker F.H., Eberly J.O., Jung C.M., Blakeney G.A., Chappell M.A. (2017). Biodegradation of insensitive munition formulations IMX101 and IMX104 in surface soils. J. Ind. Microbiol. Biotechnol..

[bib17] Kadoya W.M., Sierra-Alvarez R., Jagadish B., Wong S., Abrell L., Mash E.A., Field J.A. (2021). Covalent bonding of aromatic amine daughter products of 2,4-dinitroanisole (DNAN) with model quinone compounds representing humus via nucleophilic addition. Environ. Pollut..

[bib18] Kalderis D., Juhasz A.L., Boopathy R., Comfort S. (2011). Soils contaminated with explosives: environmental fate and evaluation of state-of-the-art remediation processes (IUPAC Technical Report). Pure Appl. Chem..

[bib19] Kirchsteiger C. (1999). On the use of probabilistic and deterministic methods in risk analysis. J. Loss Prev. Process. Ind..

[bib20] Kitcher E., Braida W., Koutsospyros A., Pavlov J., Su T.L. (2017). Characteristics and products of the reductive degradation of 3-nitro-1,2,4-triazol-5-one (NTO) and 2,4-dinitroanisole (DNAN) in a Fe-Cu bimetal system. Environ. Sci. Pollut. Control Ser..

[bib21] Krzmarzick M.J., Khatiwada R., Olivares C.I., Abrell L., Sierra-Alvarez R., Chorover J., Field J.A. (2015). Biotransformation and degradation of the insensitive munitions compound, 3-nitro-1,2,4-triazol-5-one, by soil bacterial communities. Environ. Sci. Technol..

[bib22] Lee, K. ., & Coburn, M. . (1988). Patent No. 9165. United States of America.

[bib23] Linker B.R., Khatiwada R., Perdrial N., Abrell L., Sierra-Alvarez R., Field J.A., Chorover J. (2015). Adsorption of novel insensitive munitions compounds at clay mineral and metal oxide surfaces. Environ. Chem..

[bib24] Lotufo G.R., Biedenbach J.M., Sims J.G., Chappell P., Stanley J.K., Gust K.A. (2015). Bioaccumulation kinetics of the conventional energetics TNT and RDX relative to insensitive munitions constituents DNAN and NTO in Rana pipiens tadpoles. Environ. Toxicol. Chem..

[bib25] Lu C., Samper J., Luis Cormenzana J., Ma H., Montenegro L., Ángel Cuñado M. (2012). Reactive transport model and apparent K d of Ni in the near field of a HLW repository in granite. Comput. Geosci..

[bib27] Madeira C., Field J., Simonich M., Tanguay R., Chorover J., Sierra-Alvarez R., Sierra-Alvarez R. (2018). Ecotoxicity of the insensitive munitions compound 3-nitro-1,2,4-triazol-5-one (NTO) and its reduced metabolite 3-amino-1,2,4-triazol-5-one (ATO). J. Hazard Mater..

[bib28] Mark N., Arthur J., Dontsova K., Brusseau M., Taylor S. (2016). Adsorption and attenuation behavior of 3-nitro-1,2,4-triazol-5-one (NTO) in eleven soils. Chemosphere.

[bib29] Mark N., Arthur J., Dontsova K., Brusseau M., Taylor S., Šimůnek J. (2017). Column transport studies of 3-nitro-1,2,4-triazol-5-one (NTO) in soils. Chemosphere.

[bib30] Menezes O., Kadoya W.M., Gavazza S., Sierra-Alvarez R., Mash E.A., Abrell L., Field J.A. (2021). Covalent binding with model quinone compounds unveils the environmental fate of the insensitive munitions reduced product 2,4-diaminoanisole (DAAN) under anoxic conditions. J. Hazard Mater..

[bib31] Molina, G. M., Padilla, I., Pando, M., & Pérez, D. D. (2006). Field lysimeters for the study of fate and transport of explosive chemicals in soils under variable environmental conditions. Detection and Remediation Technologies for Mines and Minelike Targets XI, 6217, 62173A.

[bib32] O’Doherty K.C., MacKenzie M.K., Badulescu D., Burgess M.M. (2013). Explosives, genomics, and the environment: conducting public deliberation on topics of complex science and social controversy. Sage Open.

[bib33] Olivares C.I., Abrell L., Khatiwada R., Chorover J., Sierra-Alvarez R., Field J.A. (2016). Bio)transformation of 2,4-dinitroanisole (DNAN) in soils. J. Hazard Mater..

[bib34] Panik M., Necas V. (2013). GOLDSIM models of long-term radiation impact of conditionally cleared radioactive material. Prog. Nucl. Energy.

[bib35] Sandham L.A., Vyver F. V. der, Retief F.P. (2013). The performance of environmental impact assessment in the explosives manufacturing industry in South Africa. J. Environ. Assess. Pol. Manag..

[bib36] Temple T., Ladyman M., Mai N., Galante E., Ricamora M., Shirazi R., Coulon F. (2018). Investigation into the environmental fate of the combined Insensitive High Explosive constituents 2,4-dinitroanisole (DNAN), 1-nitroguanidine (NQ) and nitrotriazolone (NTO) in soil. Sci. Total Environ..

[bib37] Terracciano A., Christodoulatos C., Koutsospyros A., Zheng Z., Su T.L., Smolinski B., Meng X. (2018). Degradation of 3-nitro-1,2,4-trizole-5-one (NTO) in wastewater with UV/H2O2 oxidation. Chem. Eng. J..

[bib38] Todde G., Jha S.K., Subramanian G., Shukla M.K. (2018). Adsorption of TNT, DNAN, NTO, FOX7, and NQ onto cellulose, chitin, and cellulose triacetate. Insights from Density Functional Theory calculations. Surf. Sci..

[bib39] UK Government Office for Science. (2018). Computational Modelling: Technological Futures. Retrieved from https://www.gov.uk/government/publications/computational-modelling-blackett-review

[bib40] Vila M., Lorber-Pascal S., Laurent F. (2008). Phytotoxicity to and uptake of TNT by rice. Environ. Geochem. Health.

[bib41] Vitale C.M., Di Guardo A. (2019). A review of the predictive models estimating association of neutral and ionizable organic chemicals with dissolved organic carbon. Sci. Total Environ..

[bib42] Walsh M., Gullett B., Walsh M., Bigl M., Aurell J. (2018). Improving post-detonation energetics residues estimations for the Life Cycle Environmental Assessment process for munitions. Chemosphere.

[bib43] Wei R., Fei Z., Yoosefian M. (2021). Water molecules can significantly increase the explosive sensitivity of Nitrotriazolone (NTO) in storage and transport. J. Mol. Liq..

[bib44] Won J., Borden R.C. (2017). Laboratory column evaluation of high explosives attenuation in grenade range soils. J. Environ. Qual..

[bib45] Yamamoto H., Morley M., Speitel G.E., Clausen J. (2004). Fate and transport of high explosives in a sandy soil: adsorption and desorption. Soil Sediment Contam.: Int. J..

