# Supplementary material for: Predicting the transport of 2,4-dinitroanisole (DNAN) and 3-nitro-1,2,4-triazol-5-one (NTO) in sandy and sandy loam soils
Source: Heliyon. 2022 Nov 22;8(11):e11758. doi: 10.1016/j.heliyon.2022.e11758 (PMC9694108; doi:10.1016/j.heliyon.2022.e11758)
Supplement: Supplementary material- edited - clean version [file mmc1.docx]

**Predicting the transport of 2,4-dinitroanisole (DNAN) and 3-nitro-1,2,4-triazol-5-one (NTO) in sandy and loamy soils**

Encina Gutierrez-Carazo^a^, James Dowle^b^, Frederic Coulon^c^, Tracey Temple^a^, Melissa Ladyman*^a^

^a^ Cranfield University, Centre for Defence Chemistry, Defence Academy of the United Kingdom, Shrivenham SN6 7LA, UK

^b^ Golder, Sirius Building, The Clocktower, Edinburgh, EH12 9LB, UK

^c^ Cranfield University, School of Water, Energy and Environment, Cranfield, MK43 0AL, UK

* Corresponding author

**Supplementary material**

**Supplementary 1. Scheme of the model developed in GoldSim**

## **.**
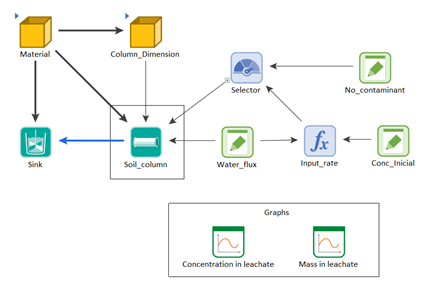


**Figure S1**. GoldSim graphical representation model for studying DNAN and NTO breakthrough in soils.

**Supplementary 2. Sensitivity analysis performed to simulated NTO breakthrough by variating soil bulk density (D), soil porosity (P) and soil tortuosity (τ).**


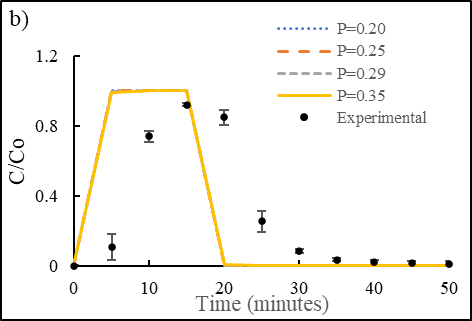

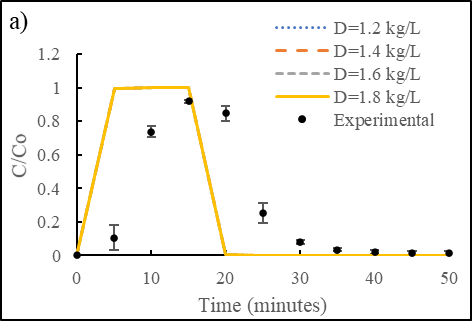

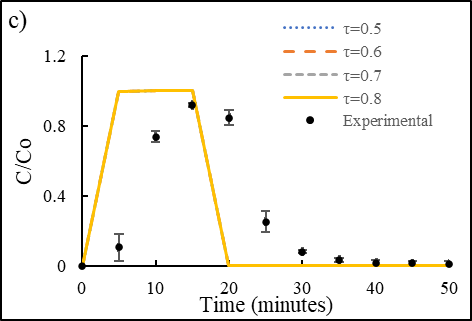


**Figure S2.** Sensitivity analysis. Variation of NTO breakthrough in sandy when modifying soil density (a), porosity (b), and tortuosity (c).
